# On the margins of aid orthodoxy: the Brazil-Mozambique collaboration to produce essential medicines in Africa

**DOI:** 10.1186/s12992-014-0070-z

**Published:** 2014-09-25

**Authors:** Giuliano Russo, Lícia de Oliveira, Alex Shankland, Tânia Sitoe

**Affiliations:** International health and biostatistics department, Instituto de Higiene and Medicina Tropical, Universidade Nova de Lisboa, Rua da Junqueira 100, Lisbon, Portugal; Centro de Malária e outras Doenças Tropicais, Instituto de Higiene e Medicina, Tropical, Lisbon, Portugal; Farmanguinhos and Fundação Oswaldo Cruz, Rio de Janeiro, Brazil; Institute of Development Studies, the University of Sussex, Brighton, UK; Departamento de Assistência Médica, Ministry of Health of Mozambique, Maputo, Mozambique

**Keywords:** AIDS, Pharmaceutical production, Aid architecture in health, Brazil pharmaceuticals, ARV, Manufacturing in Africa, Aid effectiveness in health

## Abstract

**Background:**

On the back of its recent economic development and domestic success in the fight against HIV/AIDS, Brazil is helping the Government of Mozambique to set up a pharmaceutical factory as part of its South-South cooperation programme. Until recently, a consensus existed that pharmaceutical production in Africa was not viable or sustainable. This paper looks into practicalities and evolution of this collaboration to illustrate the characteristics of Brazilian development cooperation in health, with the aim of drawing lessons for the wider debate on aid and local production of pharmaceuticals in Africa.

**Discussion:**

We show that the project process has been very long and complex, has involved multiple public and private partners, and cost in excess of USD34 million. There have also been setbacks in the process, and although production has already started, it is unclear whether all the project’s original objectives will be met.

**Summary:**

The Brazil-Mozambique’s pharmaceutical factory experience illustrates positives as well as limitations of Brazil’s unorthodox approach to health development cooperation, highlighting its contribution to pushing the boundaries of the debate on local production of pharmaceuticals in resource-poor settings.

## Background

On the back of its economic growth, newly acquired international standing and domestic public health record, Brazil has set out to expand its South-South cooperation in the health sector and in the specific field of HIV/AIDS [1]. Like other emerging donors, Brazil has not yet come to define a comprehensive development cooperation policy, nor does it subscribe to the principles of aid effectiveness [2,3]. This grants Brazil and other emerging donors unprecedented flexibility in the interpretation of their cooperation programmes, allowing for out-of-the-box thinking on modalities and features of their cooperation projects in comparison to traditional donors [4].

In their support for access to medicines in African countries, traditional donors have given priority to global purchasing and distribution initiatives rather than to domestic production. They have tended to subscribe to the consensus that African pharmaceutical factories would not be able to produce quality pharmaceuticals at competitive prices [5]. However, the African socio economic climate is rapidly changing because of the discovery of additional natural resources, changing geopolitical influences, and maturing demographic dividends [6]. Brazil is taking advantage of this context to expand its foreign and global health policy influence by undertaking bilateral cooperation projects with those countries and in those domains where it enjoys a competitive advantage, implementing a so-called ‘health diplomacy’ [7,8]. As part of this process, it is helping the government of Mozambique to set up a publicly-owned pharmaceutical factory in the outskirts of the capital city Maputo; this will also produce Antiretrovirals (ARVs), inspired by Brazil’s domestic success in the fight against HIV/AIDS, its beliefs on the connection between a country’s industrial growth and its health system development, and by strongly-held principles of economic self-reliance and state capitalism.

**Figure 1:**
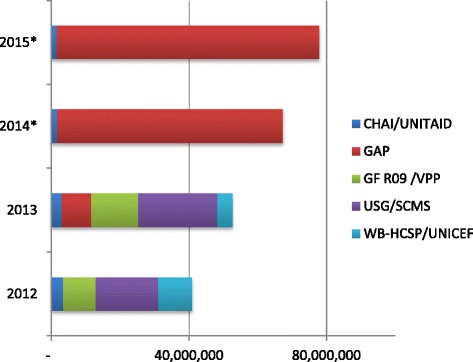
**Mozambique’s annual ARV imports, per funding agency (2012 USD).**

HIV/AIDS prevalence in Mozambique is currently estimated at 11.5% among the adult population [8]; Antiretroviral treatment was introduced in 2003 and rapidly scaled up with the support of international initiatives [9]. In 2011 an estimated 273,501 patients were receiving ARV treatment, approximately 45.5% of those needing it, and the Ministry of Health of Mozambique (MISAU) is currently planning on scaling-up ART to cover 80% of those suitable for treatment [10]. In 2012 approximately USD 66 Million worth of HIV/AIDS drugs were distributed through the public health care system, procured, funded and imported by international structures such as the Clinton Health Access Initiative (CHAI/UNITAID), USAID’s Global HIV/AIDS Programme (GAP), the UN Global Fund (GF R09/VPP), USAID’s Supply Chain Management Systems (USG/SCMS), and USAID’s HIV/AIDS Clinical Services Project. These structures procure the drugs though large annual international tenders (Figure 1).

The Government of Mozambique (GoM) currently does not contribute directly to financing and acquisition of ARVs, although all the drugs are distributed through the distribution system of MISAU’s Medicines and Medical Supplies Central Services. ARV drugs in 2012 represented 56% of the country’s public drug bill [11].

The present paper looks at innovative features, achievements and setbacks of the Brazil-Mozambique collaboration to produce essential medicines in Africa, with the objective of contributing to the debate on health aid modalities and local production of pharmaceuticals in low- and middle-income countries (LMIC). This work is based on the authors’ first-hand experience of the process of setting up the factory, on the review of diplomatic agreements and business reports, and on interviews with Brazilian and Mozambican government officials, members of the international aid community and pharmaceutical industry in Mozambique. Ethical approval was granted by the *Instituto de Higiene e Medicina Tropical*’s Ethics Committee, and informed written consent was obtained by the people interviewed.

## Discussion

### The Brazilian approach to health cooperation and pharmaceutical production

Until very recently a net aid recipient, Brazil’s cooperation programme in 2010 was estimated to be worth USD 923 million between contributions to international organisations and technical projects [12], with Mozambique as its single largest recipient country [13]. With a geographic focus on Latin America and Portuguese-speaking African countries and typically lacking grant components, Brazilian technical cooperation in health is claimed to be inspired by a set of core principles, including reciprocal ‘horizontal cooperation’ between LMICs free of traditional aid conditionalities [14], promotion of the local healthcare industry as a way to spur national health system growth and evolution – described as the development of a ‘health industrial complex’ [15] –, ‘health diplomacy’ or mutual influence of global health and foreign policy objectives [16], and ‘structuring cooperation’, a form of action aimed at strengthening within cooperating countries those institutions and people that served in Brazil’s own experience as catalysts for health systems development [17].

Brazil’s position on HIV/AIDS drugs appears in line with its support to strong Government involvement in the provision of health care services [18], underpinned by a Constitutional framework that establishes a universal citizen right to health and places a duty of health care provision on the state. The growing roles of the Ministry of Health research and training agency Fundação Oswaldo Cruz (*Fiocruz*) and its pharmaceutical arm *Farmanguinhos* – influential Government institutions behind the development of the ARV industry in Brazil – are exemplifications of the strength of this paradigm of state-led health development [19]. According to a 2008 count, Brazil’s national pharmaceutical industry included 18 public pharmaceutical labs - 8 of which registered to produce ARVs - although publicly-produced drugs only represented around 5% of the total national pharmaceutical market value, and 25% of the ARVs one [20].

In its approach to pharmaceutical production Brazil represents a notable challenge to the prevailing arguments on Trade-Related Intellectual Property Rights (TRIPS) and local production of pharmaceuticals. In 2006 two conflicting pieces of legislation were passed in Brazil, one guaranteeing free access to ARVs, and the other hampering the domestic production of cheap ARVs [21]. The Sarney Law^a^ (9313/96) aimed at guaranteeing HIV/AIDS patients the right to be treated, paving the way to scaling-up ARV treatment in the country. The same year a new national Intellectual Property Law (9279/96) sanctioned the government obligation to grant patents to protect pharmaceutical companies’ right to exclusively manufacture registered drugs, limiting the possibility for local laboratories to produce lower-cost versions of ARVs under patent [22]. Through subsequent legislation, Brazil also incorporated in its patent law the TRIPS flexibilities to mitigate the effects of patents regime in developing countries, notably compulsory licensing, experimental and bolear exemptions, parallel imports and government participation in patent application process^b^. Between 2001 and 2007, Brazil made use of the above flexibilities to obtain substantial price reductions for patented ARVs, and in the case of Efavirenz, to issue compulsory licensing [20]. Brazil is also one of the founding members, key advocate and supporter of UNITAID, an innovative patent pool mechanism granting licenses to produce drugs in developing countries against the payment of royalties [23]. Since 2012 Brazil has also engaged in Productive Development Partnerships with international manufactures, where production and technology of patented pharmaceuticals are transferred to public laboratories in exchange of the right to exclusive supply of these drugs to the Unified Health Care System at negotiated prices for an agreed time-span [24].

### The debate on production of pharmaceuticals in Africa

The issue of local production of medicines is complex and often muddled by conflicting public health and industrial policy agendas [25]. Until recently, a consensus existed that Africa-based industry would not be able to produce quality pharmaceuticals at competitive prices, and that a local critical mass of industrial and socio-economic development was required to allow the industry to survive [5]. Some authors pointed out that promoting local pharmaceutical production was unlikely to bring down high medicine prices, particularly for on-patent ARVs, as these were mostly determined by international Intellectual Property Rights rather than by local manufacturing costs [26].

These views notwithstanding, numerous pharmaceutical factories have started operations in Africa, and 38 countries in the continent are at present estimated to have pharmaceutical manufacturing entities [27]. North African countries and South Africa are reported to be home to the largest and most sophisticated factories in the continent, while smaller but still significant pharmaceutical production facilities are to be found in Ethiopia, Nigeria, Kenya, Uganda and Zimbabwe [27-31]. The majority of pharmaceutical manufacturers in Africa are reported to be small private companies primarily serving the local market, although larger publicly owned enterprises are also being established^c^. International importers and distributors are also setting up their own plants in the continent or entering joint ventures with local manufacturers^d^ [28].

As a sign of change, United Nations and African Union agencies have started projects to study the arguments and evidence around local production of medicines [32]. Recent work on the subject has showed that the evidence around the supposedly high prices of locally produced medicines is at best mixed [33,34]. Some take the view that positions may be shifting around local pharmaceutical production because of the increase in demand for medicines from the developing world coupled with increased availability of funding (especially for HIV/AIDS drugs), as well as because of the recent changes in patent rules.

### Brazil-Mozambique collaboration for the production of pharmaceuticals

The idea of a Brazil-Mozambique cooperation project to set up the first public pharmaceutical factory in Africa was agreed between the former respective presidents Luiz Inácio Lula da Silva and Joaquim Chissano in 2003, developed within Brazil’s nascent approach to ‘South-South’ cooperation in the health sector, and surviving changes of governments in Brazil as in Mozambique [3]. The initiative to set up a pharmaceutical factory in Mozambique initially had the following stated objectives: (a) to secure the ARV supply for HIV/AIDS treatment in the country; (b) to jump-start pharmaceutical generics’ manufacturing in Mozambique, enabling the fulfilment of the objectives of the national Primary Care and Pharmaceutical policies; (c) to reduce the country’s dependence on pharmaceutical donations and imports, and; (d) to contribute to the creation of local capacity for pharmaceutical production and industrial management [35]. The original cooperation agreement stated that the Government of Brazil (GoB) was going to take responsibility for the project’s staff training, for procuring equipment and raw materials, for providing technical assistance, and for designing the factory and managing the project. The Government of Mozambique would have been responsible for purchasing the physical infrastructure for the factory, for undertaking rehabilitation works, for funding the factory’s recurrent expenditures, and for buying the bulk of the factory’s pharmaceutical output. No specific deadline was defined to complete Brazil’s support, but extensions of the original 2011 agreement were to be negotiated every 3 years through Official Complementing Agreements (*Ajustes complementares*) [36].

**Figure 2:**
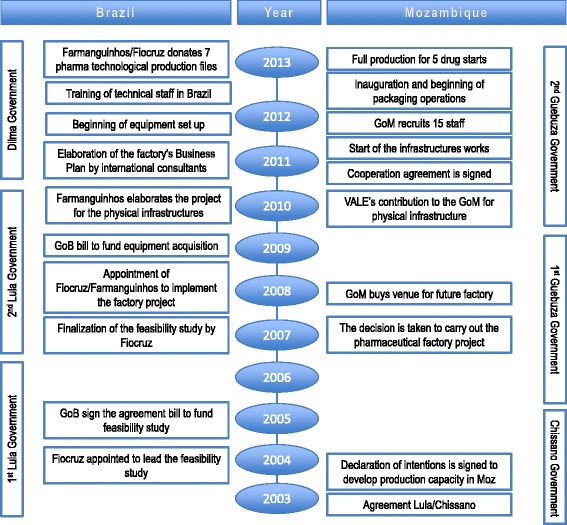
**Timelines of the implementation of the factory project.**

The first 3-year cooperation agreement was signed in 2011, and is due to be renovated through 2017. Infrastructure work was finalised in 2012, and manufacturing of a few pharmaceutical compounds (Nevirapine, Lamivudine, Captopril, Hydrochlorothiazide and Propranolol) was started in 2013 (see Figure 2).

The factory is not expected to have capacity to produce active pharmaceutical ingredients (APIs) – so-called “primary manufacturing” -, but 21 generic drugs are planned to be produced through mixing raw materials and packaging – secondary and tertiary production [5]. These include 6 ARVs, 5 hypertension drugs and a list of other drugs specifically requested by the MISAU (see full list in Table 1). A laboratory for the control of medicine quality has been already established, equipped to test drugs for efficacy and safety. When fully functional, the laboratory will be capable of providing information on the quality of all the drugs imported into the country and of contributing to the development of new drug testing methodologies.

**Table 1:** **List of pharmaceutical compounds to be produced by SMM**

**API**	**Presentation**	**Type of drug**
Amoxicilline	500 mg caps gel	Antibiotic beta-lactam
Captopryl	50 mg cmps	Antihypertensive
Captopryl	25 mg cmps	Antihypertensive
Captopryl	12.5 mg cmps	Antihypertensive
Cetoconazol	200 mg cmps	Antimycotic
Diazepam	10 mg cmps	Anxiolytic
Estavudine	40 mg cps gel	Antiretroviral
Fluconazol	100 mg cps gel	Antimycotic
Folic acid	5 mg cmp	Anti anaemic
Glibenclamide	5 mg cmps	Antidiabetic
Haloperidol	5 mg cmps	Neuroplectic
Hydrochlorthiazide	25 mg cmps	Diuretic
Lamivudine	150 mg cmp	Antiviral
Lamivudine + Zidovudine	(150 + 300) mg cmp	Antiretroviral
Lamivudine + Zidovudine + Nevirapine (adults)	(150 + 300 + 200) mg cmp	Antiretroviral
Lamivudine + Zidovudine + Nevirapine (pediatric)	(30 + 60 + 50) mg cmp	Antiretroviral
Metyldopa	500 mg cmps	Antihypertensive
Metronidazol	250 mg cmps	Anti infective
Nevirapine	200 mg cmp	Antiretroviral
Oseltamvir	75mgcps gel	Antiviral
Propranolol	40 mg cmps	Antihypertensive
Propranolol	20 mg cps gel	Antiretroviral
Propranolol	30 mg cps gel	Antiretroviral
Ribavirine	250 mg cps	Antiretroviral
Zidovudine	100 mg cps	Antiretroviral

Source: SMM Limited (2013).

Although widely known as ‘the Brazilian ARV factory’ because of its original focus on supporting the national AIDS fight and the perceived complexity of ARV production in resource-poor settings, in 2011 the enterprise was officially registered as ‘Mozambique Pharmaceutical Ltd’ (*Sociedade Moçambicana de Medicamentos*, SMM) as it plans to extend production beyond antiretroviral drugs. It is owned by the Government of Mozambique’s State Assets Management Institute (IGEPE), but the chair of its administrative board is appointed by MISAU, as is the executive director of the factory. Beside the occasional Brazilian technical assistance necessary for training and setting up the operation, two key full time *Farmanguinhos* consultants have been appointed for the next two years with the objective of steering the factory towards sustainable production and WHO Quality Certification [36].

So far the factory’s overall setup costs have been estimated at USD 34.6 million, excluding the technical assistance by Brazilian officials who are setting up the operation, which Brazil does not report as cooperation expenditures but is believed to be substantial [35]. Although the Brazilian Government funded the majority of the project’s expenditures, the Government of Mozambique contributed approximately USD 8.5 million for buying up land and existing infrastructures for the establishment of the new factory. A direct donation of USD 4.5 million from VALE, a Brazilian mining company operating in Mozambique, supported management costs and the purchase of the old infrastructures.

The factory’s business plan predicted wholesale selling price levels at which the factory would break even, on the basis of the cost structure model used for the production of ARVs in Brazil’s state pharmaceutical factories adapted to the Mozambican context [37]. Although SMM drugs face higher API costs, because of Mozambique’s burdensome import duties, as well as high maintenance costs, according to the factory’s business plan these will be offset by lower capital costs and smaller operating profits, typical of a state-subsidised company [38]. The table below shows that SMM’s ARVs unit prices compare well with those currently procured and imported by international health organizations in Mozambique and with the reference prices reported by the Management Science for Health International Price Indicator guide (Table 2).

**Table 2:** **Unit price for selected ARVs as calculated by SMM’s business plan, by source (2013 USD)**

**Product**	**SMM** ^**1**^	**CHAI** ^**2**^	**WHO** ^**2**^		**PEPFAR** ^**2**^		**MSH** ^**3**^	
			**Min**	**Max**	**Min**	**Max**	**Min**	**Max**
3TC - Lamivudine 150 mg (60 cps)	0.045	0.044	0.040	0.050	0.051	1.625	0.0408	0.1578
NVP - Nevirapine 150 mg (60 cps)	0.049	0.050	0.040	0.050	0.057	1.928	0.0452	0.2596
AZT - Zidovudine + NVP + 3TC (300 + 200 + 150 mg) (60 cps)	0.205	0.186	0.180	0.200	0.286	0.971	0.1488	0.3006
AZT - Zidovudine + 3TC (300 + 150 mg) (60 cps)	0.168	0.146	0.130	0.150	0.158	2.905	0.1446	0.4629

Source: ^1^SMM Financial Department; ^2^CMAM; ^3^Management Science for Health International Price indicator guide.

At the time of writing most of the original objectives of the factory’s project have been achieved: infrastructure works have been completed and equipment installed; 7 out of 21 technological dossiers for the production of specific pharmaceuticals have been transferred; all the permanent staff (80) bar the administrative and technical directors have been recruited and trained; production lines of intravenous solutions and 5 drugs have been started. Under the assumption that an extension to Brazilian support is agreed until 2017, the factory is expected to break even in 2016 [39].

This is the only pharmaceutical factory existing in Mozambique, and the first publicly owned in the Sub-Saharan region, although established private factories have existed for decades in South Africa, Zimbabwe and Tanzania. However, a number of issues still need to be addressed to establish the factory’s production on a sustainable path. While local skills shortages were overcome though training Mozambican staff in Brazil, the hand over of certain management positions and functions is still on going. Because of Mozambique’s poor industrial environment, all the raw materials and maintenance services for pharmaceutical transformation had to be procured abroad (mostly in South Africa, China, India and Brazil), but steady supply channels will have to be established before the likely end of Brazil’s support in 2017. Before such date international quality certifications will have to be acquired, without which there would be no access to international bids and the regional market. Given that the vast majority of the Mozambique’s public drug bill is paid by international grants and donations, whose pharmaceuticals are procured through large international tenders, it is unclear how the MISAU will find the necessary funding to absorb the factory’s output.

Providing MISAU with a reliable supply of cheap ARVs to implement its AIDS treatment strategy was one of the original objectives of the factory project. However, unlike the situation in Brazil, where the Ministry of Health is in charge of public pharmaceutical production, a public business institution – IGEPE – has been put at the helm of the state’s pharmaceutical plant in Mozambique, and MISAU’s involvement so far has been mostly limited to a supervisory role. As no mention of the factory is made in MISAU’s health policy documents, it is unclear what the GoM’s long-term plans are for the factory, including whether and in what fashion it may consider engaging in a partnership with international pharmaceutical companies to run the factory once Brazil’s support comes to an end.

## Summary

The ARV factory in Mozambique is an example of South-South cooperation in the health sector, as well as of state-driven local production of pharmaceuticals. As the factory has not started full production yet, it is difficult to accurately predict its impact on Mozambique’s pharmaceutical sector and ultimately, on its population’s access to drugs. However, a number of considerations can be drawn from this experience.

The factory represents a health sector investment of USD 34.6 million over 10 years; this represents a fairly minor cooperation project in a country that according to some estimates [40] received in excess of USD 2.5 billion of development assistance for health between 2002 and 2010. However, the Maputo factory’s potential ramifications for the Mozambican pharmaceutical market and access to ART are likely to be substantial, not least because of the interest the project has attracted in the media [41]. It seems fair to conclude that Brazil has managed to punch above the weight of its limited funds for health development cooperation with Africa by drawing from its domestic experience and expertise, and by showing a willingness to venture into unchartered cooperation modalities that are considered unorthodox by official development cooperation agencies.

The Maputo factory experience also suggests that local factories may be able to produce at competitive prices with the help of government and international cooperation subsidies. It also appears to show that a publicly owned pharmaceutical factory in LMIC would be able to generate information-related public benefits that are often ignored by the traditional arguments around local production of medicines; a public factory may enable government to regulate by participating in the pharmaceutical market, feeding back vital information on drug efficacy, costs and players’ conduct [42,43]. Traditional setbacks in development project implementation such as changes in governments, scarcity of skills, capital, services and raw materials in the local market have been overcome by, respectively, training staff in Brazil, procuring products and services from neighbouring countries, and resorting to public-private support for extra funds.

Sustainability of the factory after the likely end of Brazil’s support in 2017 remains an issue. Brazil’s original objective was to set up a pharmaceutical factory under the direct control of MISAU to help implementing its pharmaceutical policy, the way public enterprises are set up in Brazil. However, the GoM’s appointment of IGEPE, together with the conspicuous absence of references to the factory in MISAU’s policy documents, seem to signal a more pronounced interest in the factory’s contribution to the country’s industrial asset, rather than to its public health goals. The GoM will have to decide sooner rather than later whether it is still in its interest to keep the factory as a public enterprise, or to privatize it in the way similar experiences developed in Uganda and South Africa [44,45]. The lack of flexibility of the international drugs financing environment also appears to be a key limiting factor for the development of local production of pharmaceuticals in Mozambique; even if drugs were made available at competitive prices, the way external funds are currently regulated would stand on the way of procuring locally produced drugs. Furthermore, free internationally procured ARVs end up crowding out the local private sector, which is traditionally a key customer for locally produced goods [46,47].

Crucially, the setbacks experienced so far in securing the factory’s financial, technical and political sustainability expose Brazil’s lack of familiarity with the complexities of development project implementation in a context that is very different from its own [48]. Many risks have been taken in this project, from underestimating the impact of government changes in political will, to the complexity of securing public sector’s drugs purchases, and the conundrum of recruiting and retaining skilled personnel in Africa [27]. As things stand, it is still unclear whether the project will manage to achieve all its original objectives, but if it does, it will have contributed to pushing the boundaries of the debates on health aid and on local production of pharmaceuticals. Judging by the interest that the factory is attracting worldwide [49], many health policymakers, academics and aid practitioners are watching this space.

## Endnotes

^a^Named after the senator who promoted the bill.

^b^Such TRIPS flexibilities are defined as: the possibility to grant third parties the right to produce a patented drug without consent of the patent holder for public health emergencies (compulsory licensing); reproducing drugs under patent for experimental use (experimental exemption); carrying out all the required tests for drugs generic production before their patents expire (bolear exemption); importing lower-price drugs from other countries without the consent of the patent holder on the domestic market (parallel import), and; the possibility for government agencies to intervene and provide their consent in the patent application process (government participation).

^c^Such as Saidal in Algeria and Saphad in Tunisia.

^d^For example CIPLA and Quality Pharmaceuticals in Uganda.

## References

[1] The Economist: **Brazil’s Foreign-Aid Programme: Speak Softly and Carry a Blank Cheque.** In *The Economist.* 2010. Available from: http://www.economist.com/node/16592455.

[2] Harmer A, Xiao Y, Missoni E, Tediosi F (2013). “BRICS without straw”? A systematic literature review of newly emerging economies’ influence in global health. Glob Health.

[3] Russo G, Cabral LV, Ferrinho P (2013). Brazil-Africa technical cooperation in health: what’s its relevance to the post-Busan debate on ’aid effectiveness. Glob Health.

[4] Kragelund P (2008). The return of non-DAC Donors to Africa: New prospects for African development?. Dev Policy Rev.

[5] Kaplan AW, Laing R: **Local Production of Pharmaceuticals: Industrial Policy and Access to Medicines. An Overview of Key Concepts, Issues and Opportunities for Future Research.** In *International Bank for Reconstruction and Development/The World Bank*, HPN Discussion Paper 32036. Washington: 2005.

[6] Eastwood R, Lipton M (2012). The demographic dividend: retrospect and prospect. Econ Aff.

[7] Russo G, Shankland A (2013). Brazil’s engagement in health co-operation: what can it contribute to the global health debate?. Health Policy Plan.

[8] INS INE, Macro ICF (2010). Inquérito Nacional de Prevalência, Riscos, Comportamentais e Informação sobre o HIV e SIDA em Moçambique 2009 (INSIDA).

[9] GoM: *National Strategic HIV and AIDS Response Plan, 2010 – 2014*, Republic of Mozambique, Council of Ministers. 2009.

[10] GoM: *Plano de Aceleração da Resposta ao HIV e SIDA em Moçambique 2013–2015*, The Ministry of Health of Mozambique. 2013.

[11] COWI (2012). Finalização do plano de negócios da SMM: Situação dos Mercados Farmacêuticos em Moçambique e na Região da SADC.

[12] IPEA (2011). Cooperaçao Brasileira para o Desenvolvimento Internacional 2005–2009.

[13] Saggioro Garcia A, Martins Kato KH (2014). A história da caça ou do caçador? Reflexões sobre a inserção do Brasil na África. World Tens.

[14] ECOSOC (2008). Report of the first development cooperation forum [Internet].

[15] Gadelha CAG (2006). Development, health-industrial complex and industrial policy. Rev Saude Publica.

[16] Kickbusch I, Silberschmidt G, Buss P (2007). Global health diplomacy: the need for new perspectives, strategic approaches and skills in global health. Bull World Health Organ.

[17] Buss P (2011). Brazil: structuring cooperation for health. Lancet.

[18] Victora CG, Barreto ML, DO Carmo Leal M, Monteiro CA, Schmidt MI, Paim J, Bastos FI, Almeida C, Bahia L, Travassos C, Reichenheim M, Barroso F (2011). Health conditions and health-policy innovations in Brazil: the way forward. Lancet.

[19] Homedes N, Ugalde A (2006). Improving access to pharmaceuticals in Brazil and Argentina. Health Policy Plan.

[20] Flynn M: **Corporate power and state resistance: Brazil’s use of TRIPS flexibilities for its National AIDS program.** In *Intellectual property, pharmaceuticals, and public health: access to drugs in developing.* [S.l.]: Edward Elgar Pub; 2010.

[21] Shadlen KC, Fonseca E (2013). Health policy as industrial policy: Brazil in comparative perspective. Polit Soc.

[22] Chaves GC, Vieira MF, Reis R (2008). Access to medicines and intellectual property in Brazil: reflections and strategies of civil society. Sur Rev Int Direitos Hum.

[23] Bermudez JT, Hoen E (2010). The UNITAID patent pool initiative: bringing patents together for the common good. Open AIDS J.

[24] Ministério da Saúde: *Portaria N*^*o*^*837 - Define as Diretrizes e os Critérios para o Establecimento das Parcerias Para O Desenvolvimento Produtivo (PDP) [Internet].*. Portaria 837 de 18 de Abril de 2012 2012. Available from: http://bvsms.saude.gov.br/bvs/saudelegis../gm/2012/prt0837_18_04_2012.html.

[25] Kaplan AW (2011). Local Production and Access to Medicines in Low- and Middle-Income Countries: A Literature Review and Critical Analysis.

[26] Rovira: **Creating and Promoting Doemstic Drug Manufacturing Capacities: a Solution for Developing Countries? Negotiating Health: Intellectual Property and Access to Medicines.** In *International Centre for Trade and Sustainable Development.*Sterling, VA: Earthscan; 2006.

[27] UNIDO-AUC (2012). Pharmaceutical Manufacturing Plan for Africa - Business Plan.

[28] UNIDO (2010). Pharmaceutical Sector Profile: Uganda.

[29] UNIDO (2010). Pharmaceutical Sector Profile: Kenya.

[30] UNIDO (2011). Pharmaceutical Sector Profile: Nigeria.

[31] UNIDO (2011). Pharmaceutical Sector Profile: Zimbabwe.

[32] WHO (2011). Local Production for Access to Medical Products: Developing a Framework to Improve Public Health.

[33] Mackintosh M, Chaudhuri S, Mujinja PG (2011). Can NGOs regulate medicines markets? Social enterprise in wholesaling, and access to essential medicines. Glob Health.

[34] Kuanpoth J (2007). Patents and access to antiretroviral medicines in Vietnam after world trade organization accession. J World Intellect Prop.

[35] De Oliveira L (2013). Inicitativa de Instalação da Fábrica de Antiretrovirrais e Outros Medicamentos Em Moçambique; Avaliação do Projecto.

[36] De Oliveira L (2012). Nota Informativa Sobre a Iniciativa de Instaliação da Fábrica de Antiretrovirrais e Outrs Medicamentos em Moçambique.

[37] Pinheiro E, Vasan A, Kim JY, Lee E, Guimier JM, Perriens J (2006). Examining the production costs of antiretroviral drugs. AIDS.

[38] MacDonald R, Yamey G (2001). The cost to global health of drug company profits. West J Med.

[39] Farmanguinhos: *Plano de Negócios da SMM.* 2013.

[40] Van de Maele N, Evans DB, Tan-Torres T (2013). Development assistance for health in Africa: are we telling the right story?. Bull World Health Organ.

[41] The Mail and Guardian: *Govt Mulls State Pharmaceutical Company [Internet]*, The M&G Online. 2011. [cited 2013 Jul 30]. Available from: http://mg.co.za/article/2011-07-19-govt-mulls-state-pharmaceutical-company/.

[42] Sertel PMR (1988). Regulation by participation. J Econ.

[43] De Fraja G, Delbono F (1989). Alternative strategies of a public enterprise in oligopoly. Oxf Econ Pap.

[44] Rajagopal D: **Cipla Completes the Medpro Deal, Buys 100% Stake in the South African Company [Internet].** In *The Economic Times.* 2013. [cited 2013 Sep 26]. Available from: http://articles.economictimes.indiatimes.com/2013-02-28/news/37352125_1_cipla-medpro-cipla-chairman-subhanu-saxena.

[45] World News: *Cipla ups Stake in Ugandan Firm, Acquires 14.5% for $15 Million [Internet]*, World News. 2013. [cited 2014 Feb 5]. Available from: http://article.wn.com/view/2013/11/21/Cipla_ups_stake_in_Ugandan_firm_acquires_145_for_15_million_l/.

[46] Herzer D, Grimm M (2012). Does foreign aid increase private investment? Evidence from panel cointegration. Appl Econ.

[47] Rajan RG, Subramanian A (2011). Aid, Dutch disease, and manufacturing growth. J Dev Econ.

[48] Cabral L: *Brazil’s Development Cooperation with the South: a Global Model in Waiting [Internet]*, Overseas Development Institute (ODI). 2010. [cited 2013 Jul 11]. Available from: http://www.odi.org.uk/opinion/4952-brazils-development-cooperation-south-global-model-waiting.

[49] Cabral L, Russo G, Weinstock J (2014). Brazil and the shifting consensus on development co-operation. Dev Policy Rev.

